# Serdexmethylphenidate/dexmethylphenidate for children with attention-deficit/hyperactivity disorder: dose optimization from a laboratory classroom study

**DOI:** 10.3389/fpsyt.2024.1310483

**Published:** 2024-03-19

**Authors:** Andrew J. Cutler, Scott H. Kollins, Matthew N. Brams, Meg Corliss, Charles Oh, Rene Braeckman, Ann C. Childress

**Affiliations:** ^1^ State University of New York (SUNY) Upstate Medical University, Syracuse, NY, United States; ^2^ Neuroscience Education Institute, Lakewood Ranch, FL, United States; ^3^ Duke University School of Medicine, Durham, NC, United States; ^4^ Akili Interactive, Inc., Boston, MA, United States; ^5^ Bayou City Research, Houston, TX, United States; ^6^ Corium LLC, Boston, MA, United States; ^7^ Zevra Therapeutics Inc., Celebration, FL, United States; ^8^ Center for Psychiatry and Behavioral Medicine, Las Vegas, NV, United States

**Keywords:** ADHD, ADHD-RS-5, Azstarys, SDX/d-MPH, dose optimization, Conners 3-P

## Abstract

**Objective:**

To evaluate treatment responder rate using the Attention-Deficit/Hyperactivity Disorder Rating Scale-5 (ADHD-RS-5) score based on optimized dose level of serdexmethylphenidate/dexmethylphenidate (SDX/d-MPH) and changes in ADHD severity in children (aged 6–12 years) with ADHD.

**Methods:**

During a 21-day dose-optimization phase, 155 patients initiated treatment with 39.2/7.8 mg SDX/d-MPH in the first week and then were titrated to an optimum dose; 5 patients were downtitrated to 26.1/5.2 mg, 76 were uptitrated to 52.3/10.4 mg, and 69 remained at 39.2/7.8 mg during the following 2 weeks. Responder threshold values were 30% and 50% based on the percent change from baseline (day 0) to days 7, 14, and 21 in the ADHD-RS-5 score. The Conners 3rd Edition-Parent score was used to assess weekly changes in ADHD severity during the dose-optimization and treatment phases.

**Results:**

Of the 5 subjects whose dose was optimized at 26.1/5.2 mg, ≥80% across all days had ≥50% responder rate. Of the 69 subjects whose dose was optimized at 39.2/7.8 mg, 81.2% had ≥50% responder rate by day 21. Of the 76 subjects whose dose was optimized to 52.3/10.4 mg, 72.4% had ≥50% responder rate by day 21. Changes in ADHD severity, based on mean Conners 3rd Edition-Parent scores, improved from baseline at each visit during dose optimization for each subscale. At the dose-optimization phase, Conners 3rd Edition-Parent scores improved from baseline for SDX/d-MPH in all subscales.

**Conclusion:**

A high percentage of subjects were responders upon reaching their final optimized dose. SDX/d-MPH demonstrated significant reductions in ADHD severity in children based on the Conners 3rd Edition-Parent scores. Determining the optimal dosage of SDX/d-MPH and its effect on ADHD severity could enable the development of a more clinically relevant treatment regimen in children with ADHD.

## 1 Introduction

Attention-deficit/hyperactivity disorder (ADHD) is a common neurobehavioral disorder that occurs in 9.8% of children in the United States (1) and has a worldwide prevalence ranging from approximately 5% to 7% (2–4), with more than 90% of diagnoses persisting into adulthood (5). People with ADHD show characteristic symptoms of inattention, hyperactivity, and impulsivity, which can adversely affect their behavioral, social, emotional, academic, occupational, and executive functioning (6).

Methylphenidate (MPH) is a psychostimulant medication that is commonly prescribed to treat ADHD because of its effectiveness in reducing symptoms (7). Despite availability of various once-daily MPH products, children, parents, and caregivers have reported inconsistent ADHD symptom control while taking the medications (8). The duration of treatment effect is of particular concern; often these medications are given to show effect within the classroom, even though the effect needs to extend beyond the school hours to improve overall quality of life for children, parents, and caregivers (8). Thus, a treatment that provides onset within 30 minutes and sustained symptom control for up to 13 hours is an unmet medical need.

Serdexmethylphenidate/dexmethylphenidate (SDX/d-MPH; Azstarys^®^, Corium LLC, Boston, MA, USA) is a once-daily, oral treatment approved for patients aged ≥6 years with ADHD. SDX/d-MPH contains 70% SDX, a novel prodrug of d-MPH, and 30% d-MPH. After oral administration, early symptom control is provided by the d-MPH component, whereas mid- to late-day symptom control is governed by gradual conversion of SDX to active d-MPH in the lower intestinal tract.

The efficacy of SDX/d-MPH was demonstrated in a 4-week pivotal laboratory classroom study of children aged 6 to 12 years, in which a significant improvement in ADHD symptoms was observed compared with placebo, with a rapid onset and extended duration of treatment effect. The treatment was well tolerated, with a safety profile similar to that of other stimulant treatments (9). In the primary efficacy analysis, SDX/d-MPH significantly improved from baseline the Swanson, Kotkin, Agler, M-Flynn, and Pelham combined (SKAMP-C) scores averaged across the laboratory classroom day versus placebo. The treatment effect for SDX/d-MPH versus placebo was observed from 0.5 to 13 hours postdose.

Dose titration/optimization is important with pharmacological treatments for ADHD, as it ensures that there is an optimal balance between treatment effectiveness and safety and tolerability of that treatment (7). ADHD treatment guidelines in the United States and worldwide highlight the need to evaluate dose titration and optimization of stimulant treatments. Dosage optimization (DO) for stimulant treatments is required to achieve the greatest benefit with the fewest number of tolerable side effects (10).

In the pivotal laboratory classroom study of SDX/d-MPH, before evaluation of the primary efficacy end point, SDX/d-MPH dose for each subject was optimized based on maximal efficacy and tolerability in a 3-week, open-label DO phase (9). The goal of this *post hoc* analysis was to evaluate the effect of SDX/d-MPH during the DO phase. We conducted a *post hoc* analysis to measure treatment responder rates using the ADHD-Rating Scale-5 (ADHD-RS-5) score according to the optimized dose level of SDX/d-MPH. The ADHD-RS-5 rating scale rates the 18 symptoms associated with ADHD from the Diagnostic and Statistical Manual of Mental Disorders, 5th edition (DSM-5) (11). We also evaluated the effects of SDX/d-MPH on ADHD severity using the Conners 3rd Edition-Parent (Conners 3-P) in an exploratory analysis of the study. Conners 3-P is a parent- and caregiver-reported outcome that provides a detailed evaluation of the severity of ADHD symptoms (12).

## 2 Methods

### 2.1 Study design

**Figure 1:**
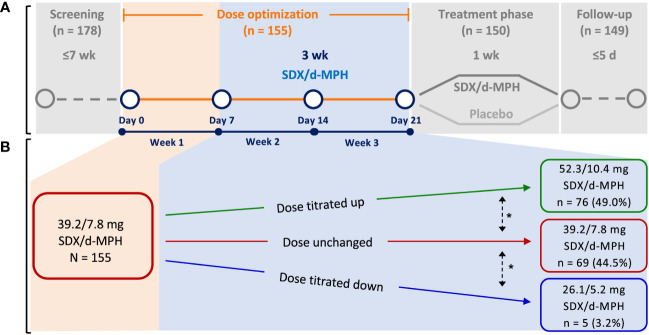
Study design **(A)** and dose titration **(B)**. SDX/d-MPH, serdexmethylphenidate/dexmethylphenidate. *Dose could be further titrated up or down as needed.

These analyses included data from a multicenter, dose-optimized, double-blind, randomized, placebo-controlled, parallel-efficacy laboratory classroom study in children with ADHD aged 6 to 12 years (NCT03292952). For diagnosis of ADHD and inclusion into the study, subjects must have met DSM-5 criteria for a primary diagnosis of ADHD (combined, inattentive, or hyperactive/impulsive presentation) per clinical evaluation and confirmed by the Mini International Neuropsychiatric Interview for Children and Adolescents, must have had a score of at least 3 (mildly ill) on the clinician-administered Clinical Global Impressions-Severity scale, and must have had an ADHD-RS-5 total score of at least 28 at Day 0 of the study. Subjects were excluded if they had other psychiatric or central nervous system disease diagnoses, or suicidal ideation or a history of suicide attempt (9). This study was conducted across 5 study centers located in the United States. The study was initiated on December 20, 2017, and the last follow-up visit occurred on May 16, 2018. The study design has been published previously (9) and consisted of a 3-week, open-label DO phase followed by a 1-week dose-optimized, blinded, randomized, controlled treatment phase (**Figure 1A**). During the DO phase, subjects started treatment at 39.2/7.8 mg SDX/d-MPH and were titrated to an optimal SDX/d-MPH dose of 26.1/5.2 mg, 39.2/7.8 mg, or 52.3/10.4 mg based on maximal efficacy and tolerability in the opinion of the investigator (**Figure 1B**). The investigator’s consideration for SDX/d-MPH dosage adjustment was made based on a ≥30% reduction (showing improvement) in ADHD-RS-5, improvement in the Clinical Global Impressions–Improvement scores, interview with the parent or caregiver, and safety data of subjects at the end of weeks 1 and 2 of the DO phase. During the subsequent 1-week treatment phase, subjects received once-daily SDX/d-MPH at their optimized-dose levels or placebo. SDX/d-MPH 26.1/5.2-mg, 39.2/7.8-mg, and 52.3/10.4-mg doses correspond with 20-, 30-, and 40-mg molar equivalents, respectively, of total d-MPH. Subjects were assessed approximately 3 days after administration of the last dose of the treatment phase to assess safety parameters. The primary objective of the study was to determine the efficacy of SDX/d-MPH versus placebo. The primary end point was mean change from baseline in the SKAMP-C scores.

### 2.2 DO phase ADHD-RS-5 responder rate analysis

In the first week of the DO phase, 155 enrolled subjects started their treatment with the 39.2/7.8-mg dose (**Figure 1B**). During the subsequent 2 weeks, subjects were titrated to an optimum dose (1 of 3 available doses) based on physician assessment. Seventy-six subjects (49.0%) were uptitrated to 52.3/10.4 mg. Sixty-nine subjects (44.5%) remained at the 39.2/7.8-mg dose level during the following 2 weeks. Five subjects (3.2%) were downtitrated to 26.1/5.2 mg.

The level of response was evaluated at days 7, 14, and 21 by the optimized-dose levels that were achieved at the end of the DO phase using the ADHD-RS-5 score collected during this phase. ADHD-RS-5 is an 18-item scale based on the DSM-5 criteria (6) for ADHD that rates symptoms on a 4-point scale. ADHD-RS-5 scores were obtained during a clinician-directed interview with the parent/guardian/caregiver. The analysis involved defining a threshold value equal to or above which a subject was considered a “responder” and below which a subject was considered a “nonresponder.” Two responder variables were created, with threshold values of 30% and 50% based on the percent improvement in the ADHD-RS-5 score from baseline (day 0) to days 7, 14, and 21. Whereas 30% improvement has traditionally been used for response, 50% improvement has been shown to be more clinically relevant (13, 14).

### 2.3 Conners 3-P exploratory analysis

Weekly changes from baseline in Conners 3-P score were assessed for the DO and treatment phases. Conners 3-P is a 43-item parent, guardian, or caregiver report that evaluates severity of ADHD symptoms using 6 assessment subscales: inattention, hyperactivity/impulsivity, learning problems, executive functioning, aggression, and peer relationships (12). Each item was scored on a 4-point scale ranging from “not true at all (never, seldom)” to “very much true (very often, very frequently).”

### 2.4 Statistical analyses

The analyses were performed using the intent-to-treat population, defined as all randomized subjects who received ≥1 dose of open-label study medication and ≥1 dose of double-blind study medication and had ≥1 postdose SKAMP-C assessment at day 28. For the ADHD-RS-5 responder analysis by the optimized-dose level, frequencies and percentages of subjects within the 30% and 50% threshold levels were calculated for days 7, 14, and 21. A χ^2^ test was used to compare responder rate differences between dose levels. The differences in Conners 3-P score changes from baseline between SDX/d-MPH and placebo at the end of the treatment phase were assessed using a mixed-effect model for repeated measures (significance level of 0.05), and the differences between baseline and each visit of the DO phase were evaluated using a paired *t*-test.

## 3 Results

### 3.1 Subjects

Subject disposition has been described previously in detail (9). Of 155 subjects enrolled in the open-label DO phase, 150 continued to the double-blind, randomized, placebo-controlled treatment phase; 5 subjects discontinued after the DO phase, 4 because of a treatment-emergent adverse event, and 1 because of failure to meet randomization criteria. All randomized subjects received ≥1 dose of double-blind study drug. Demographics and baseline characteristics of the subjects who were randomized after the DO phase have been described previously (9) and are shown in **Table 1**. Mean age of the subjects was 9.6 years, and subjects were predominantly male (61.3%), White (50.7%) or Black/African American (37.3%), and not Hispanic or Latino (73.3%; **Table 1**).

**Table 1:** Subject demographics and baseline characteristics (intent-to-treat population) (6).

Parameter	Subjects (*N* = 150)
Age, y	9.6 (1.6)
Sex, ***n*** (%) Male Female	92 (61.3) 58 (38.7)
Ethnicity, ***n*** (%) Hispanic or Latino Not Hispanic or Latino	40 (26.7) 110 (73.3)
Race, ***n*** (%) White Black/African American Multiracial Asian Other	76 (50.7) 56 (37.3) 10 (6.7) 7 (4.7) 1 (0.7)
Weight, kg	39.3 (13.8)
Height, cm	140.4 (10.9)
Body mass index, kg/m^2^	19.5 (4.7)
ADHD-RS-5, overall score	41.8 (7.0)
CGI-S score	4.9 (0.8)

Values shown are mean (standard deviation) unless otherwise noted.

ADHD-RS-5, Attention-Deficit/Hyperactivity Disorder-Rating Scale-5; CGI-S, Clinical Global Impressions-Severity; SDX/d-MPH, serdexmethylphenidate/dexmethylphenidate.

### 3.2 DO responders

**Figure 2:**
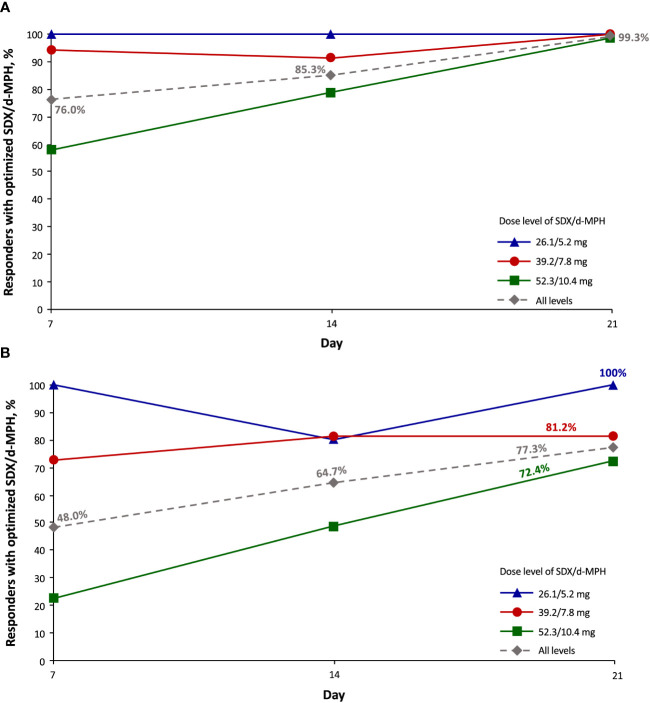
Responder rate of ≥30% **(A)** and ≥50% **(B)** as assessed with the Attention-Deficit/Hyperactivity Disorder Rating Scale-5 (intent-to-treat population). SDX/d-MPH, serdexmethylphenidate/dexmethylphenidate.

Nearly all subjects (99.3%) at all 3 final optimized-dose levels had a ≥30% responder rate by day 21 (**Figure 2A**). By day 21, 77.3% of subjects had a ≥50% responder rate (**Figure 2B**). Of  76 subjects whose dose was optimized to the 52.3/10.4-mg dose (initiated at the 39.2/7.8-mg dose), 22.4% had a ≥50% responder rate at day 7, and 72.4% had a ≥50% responder rate by day 21. Of the 69 subjects whose dose was optimized at the 39.2/7.8-mg dose, 72.5% had a ≥50% responder rate at day 7, and 81.2% had a ≥50% responder rate by day 21. Of the 5 subjects whose dose was optimized at the 26.1/5.2-mg dose (initiated at the 39.2/7.8-mg dose), ≥80% of subjects across all visit days had a ≥50% responder rate.

### 3.3 Conners 3-P scores

**Figure 3:**
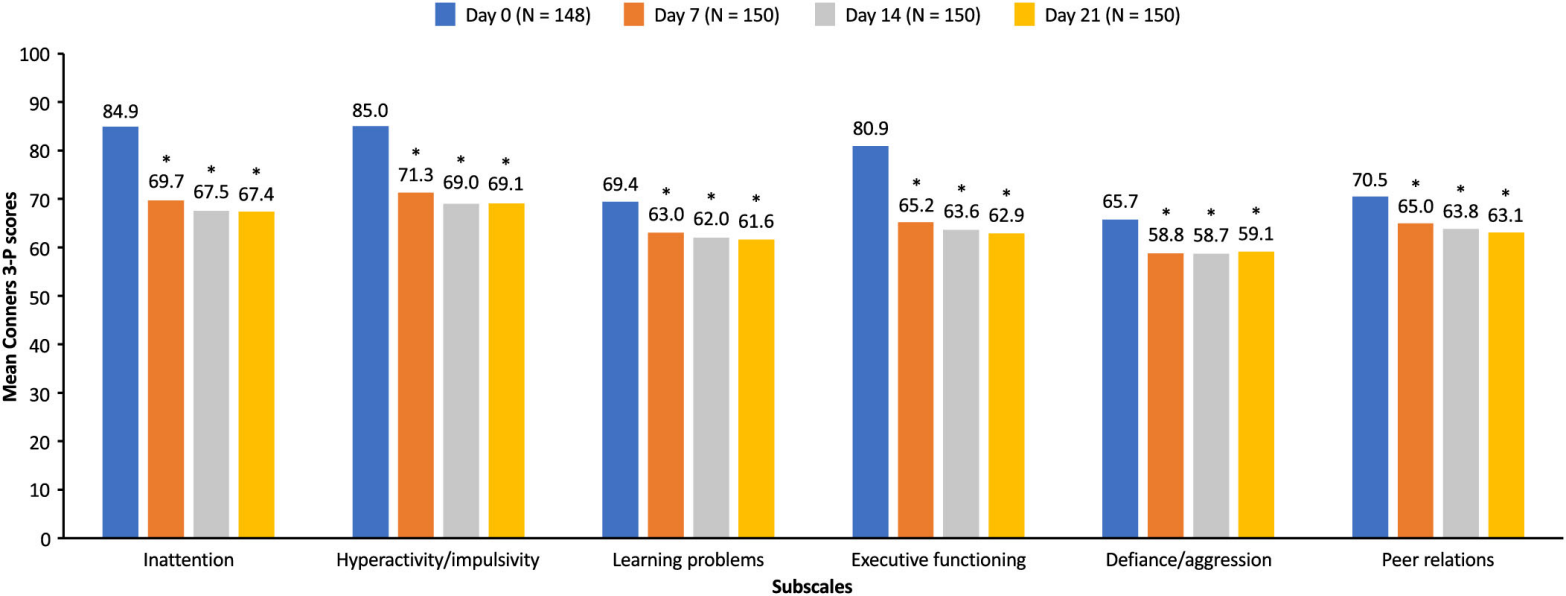
Mean Conners 3-P scores during the open label dose-optimization phase (intent-to-treat population). Mean Conners 3-P scores for each subscale were measured at day 0 and at each subsequent visit (days 7, 14, and 21) during the open-label dose-optimization phase with serdexmethylphenidate/dexmethylphenidate. *p <.001 compared with score at day 0. Conners 3-P, Conners 3rd Edition-Parent.

At every study visit (days 7, 14, and 21) during the open-label DO phase, significant improvements (*p* <.001) from baseline (day 0) were observed in the mean Conners 3-P score for all subscales, including inattention, hyperactivity/impulsivity, learning problems, executive functioning, defiance/aggression, and peer relations with SDX/d-MPH (**Figure 3**). As reported previously (9), SDX/d-MPH was well tolerated and had no concerning safety signals.

## 4 Discussion

In this *post hoc* analysis of the double-blind, randomized, placebo-controlled, laboratory classroom study of children aged 6 to 12 years with ADHD, we show that the response to SDX/d-MPH treatment can be seen during the first week of treatment. ADHD-RS-5 responders were evident at the 26.1/5.2-mg and 39.2/7.8-mg final dose levels, with small increases in the proportion of responders thereafter. The proportion of responders continued to notably increase after titration to the 52.3/10.4-mg final dose level. We note that from a methodological standpoint, the goal of this *post hoc* analysis was to determine the responder rates of the 3 approved SDX/d-MPH dosages on ADHD symptoms during the DO phase of the pivotal 4-week efficacy study of SDX/d-MPH and was not designed or powered to be a stand-alone DO study. However, the findings from this analysis of treatment responder rates will be of value to clinicians because they may help in determining the optimal dose of SDX/d-MPH for the most favorable clinical effect. Those subjects having a suboptimal response to the initial 39.2/7.8-mg dose responded well to the 52.3/10.4-mg dose during the subsequent 2 weeks and achieved a ≥50% responder rate in a high percentage of those subjects. Because of concerns over safety and tolerability, stimulants are often underdosed, so our findings suggest that increasing the dose of SDX/d-MPH can be used to achieve maximum efficacy and tolerability (15).

Our results are consistent with those of other studies of MPH medications. In a placebo-controlled, crossover study of children (aged 5 to 16 years) with ADHD combined type who were treated with a long-acting osmotic-release oral system MPH, a linear dose-response relationship was observed, with a clinically significant reduction in ADHD-RS-IV scores with higher doses of osmotic-release oral system MPH compared with the lower dose (16). A linear dose-response pattern on behavioral and cognitive measures has also been observed in several studies using mild-to-moderate doses of immediate-release MPH (17–20).

In an exploratory analysis, SDX/d-MPH demonstrated significant reductions in ADHD severity versus placebo in children aged 6 to 12 years, based on the Conners 3-P subscale scores for inattention, hyperactivity/impulsivity, learning problems, and executive functioning. Significant reduction from baseline could be seen in all Conners 3-P subscale scores (inattention, hyperactivity/impulsivity, learning problems, executive functioning, defiance/aggression, and peer relations) in the DO phase, suggesting an early effect of treatment. The effectiveness of SDX/d-MPH in treating symptoms of ADHD was previously shown using the SKAMP rating scale and Permanent Product Measure of Performance (9). Results of a long-term safety study (12 months) in children aged 4 to <6 years after treatment optimization showed that symptom control was maintained on the extended-release MPH (21). The result from our study is consistent with those findings, as the Conners scales showed maintenance of symptom control (21). In addition, similar results were found in children aged 6.4 to 17.5 years, as significant improvements were reported on all 4 subscales of Conners Parent and Teacher Rating Scales when using twice-daily immediate-release MPH and extended-release MPH (22).

Our data, using 2 measures of ADHD symptoms (ADHD-RS-5 and Conners 3-P), show that the effects of SDX/d-MPH on mitigating ADHD symptoms start early during the first week of treatment. Improvement in overall symptoms were observed using both scales, but Conners 3-P data provided more details on improvements in specific symptoms of ADHD. The results of the study show that determining the optimal dose of SDX/d-MPH and its effect on ADHD severity could enable the development of a more clinically relevant treatment regimen in children with ADHD.

## 5 Limitations

The limitations of the study include a relatively short double-blind treatment phase, although it was consistent with that of similar studies. The eligibility criteria excluded children with comorbidities, thereby potentially limiting the findings.

## 6 Conclusions

In this study, response to SDX/d-MPH was evident after the first week of treatment. Dose-optimized SDX/d-MPH demonstrated statistically significant reductions in ADHD severity in children with ADHD aged 6 to 12 years.

## Data availability statement

The raw data supporting the conclusions of this article will be made available by the authors, upon written request.

## Ethics statement

The studies involving humans were approved by Copernicus Group IRB, 5000 Centre Green Way, Suite 200, Cary, NC 27513, and Duke Health IRB, Hock Plaza, Suite 405, 2424 Erwin Road, Durham, NC 27705. The studies were conducted in accordance with the local legislation and institutional requirements. Written informed consent for participation in this study was provided by the participants’ legal guardians/next of kin.

## Author contributions

AJC: Writing – original draft, writing – review & editing. SHK: Writing – original draft, writing – review & editing. MNB: Writing – original draft, writing – review & editing. MC: Writing – original draft, writing – review & editing. CO: Writing – original draft, writing – review & editing. RB: Writing – review & editing. ACC: Writing – original draft, writing – review & editing.
